# Alcohol-associated liver disease and behavioral and medical cofactors: unmet needs and opportunities

**DOI:** 10.3389/fpubh.2024.1322460

**Published:** 2024-04-04

**Authors:** Mollie A. Monnig, Hayley Treloar Padovano, Peter M. Monti

**Affiliations:** ^1^Center for Alcohol and Addiction Studies, Brown University, Providence, RI, United States; ^2^Center for Addiction and Disease Risk Exacerbation, Brown University, Providence, RI, United States; ^3^Department of Psychiatry and Human Behavior, Brown University, Providence, RI, United States

**Keywords:** liver health, integrated behavioral healthcare, alcohol-associated liver disease, chronic liver disease, alcohol use, HIV infection

## Abstract

Chronic liver disease is a leading cause of death in the US and is often preventable. Rising burden, cost, and fatality due to liver disease are driven by intensified alcohol use in the US population and the contributions of comorbid conditions. This mini-review focuses on the topic of liver health in the context of chronic, behavioral cofactors of disease, using research-based examples from the Brown University Center for Addiction and Disease Risk Exacerbation (CADRE). Our aim is to illustrate the current challenges and opportunities in clinical research addressing liver health in the context of behavioral and medical comorbidity and to highlight next steps in this crucial area of public health research and clinical care.

## 1 Introduction

Liver health is an unmet medical need globally (1, 2). Chronic liver disease is a leading cause of death in the US and worldwide and is often preventable (3–5). Alcohol-associated liver disease (ALD) is the most prevalent chronic liver disease worldwide and the leading cause for liver transplantation in the US (6). ALD is a health sequela of chronic excessive alcohol use that progresses from hepatic steatosis to alcoholic steatohepatitis and, ultimately, to fibrosis, cirrhosis, and/or hepatocellular cancer. Only a relatively small proportion of individuals progress to the final stage, with advancement influenced by biological sex, ethnicity, smoking status, obesity, and other factors (7). Early stages of liver disease may be “silent” or asymptomatic, and the disease often presents late with fatal complications (8, 9).

The mortality rate for ALD has risen in recent years, with no new treatments introduced in decades (4). ALD affects 1 in 20 US adults, and ALD-related mortality has risen sharply since 2020 (10, 11). Each year, more people are dying of ALD and at younger ages. Deaths due to alcoholic cirrhosis increased more than threefold from 1999 to 2019 (12). The increase in mortality due to alcoholic cirrhosis from 2009 to 2016 was driven by a 10.5% rise in deaths among ages 24–35 (13). Biological females and individuals of Hispanic or Latine ethnic background are disproportionately affected at lower levels of alcohol consumption (7).

At-risk individuals who eventually die of ALD have multiple interactions with medical care providers and hospitalizations in the years preceding their death, each one representing a missed opportunity for detection and intervention (14). Expert recommendations to interrupt the course of liver disease progression include: (1) strengthening detection of early liver disease in primary care to interrupt its course; (2) improving resources for community screening of at-risk individuals; (3) promoting healthy lifestyles to reduce alcohol consumption through government restrictions on alcohol sales (15). Without multidisciplinary research and intervention design, however, the problem of silently advancing chronic liver disease will remain unchecked.

The goal of the Brown University Center for Addiction and Disease Risk Exacerbation (CADRE), a National Institute of General Medical Sciences Center of Biomedical Research Excellence (P20GM130414; PI: Monti), is to examine biobehavioral mechanisms by which substance use increases risk for, and progression of, chronic disease (16). The CADRE is a thematically linked, state-of-the-art, multidisciplinary center utilizing a range of research methods, including experimental laboratory, ecological momentary assessment, health services, and computational modeling approaches. Up to half of all early deaths in the U.S. are preventable, and the leading behavioral correlates of chronic health conditions include tobacco use, unhealthy dietary patterns, alcohol intake, physical inactivity, and sex risk behaviors. These individual determinants account for approximately twice the variance in population health outcomes as direct clinical care (17). In particular, the intersection of these preventable disease cofactors compounds downstream health risks, yet linkages of substance use to chronic disease are complex and often unclear.

Thus, the CADRE thematically investigates interlocking processes by which substance use and chronic health conditions interact to increase risk of morbidity and mortality. The CADRE’s mission is realized through synergistic, innovative investigative teams with complementary expertise. CADRE investigators and affiliated scientists are united from behavioral and social sciences, psychiatry and human behavior, medicine, infectious disease, pathology and laboratory medicine, behavioral medicine, and neuroscience. Exemplar current and completed projects in the CADRE’s thematic scope include exploration of cannabis effects on pain and inflammatory biomarkers among patients with rheumatoid arthritis (subproject 5264; Project Leader: Aston), syndemic effects of tobacco smoking and alcohol use on cardiovascular disease in incarcerated individuals (Project Leader: Khanna), utilization of individualized biobehavioral feedback for alcohol use disorder (AUD) to reduce ALD risk and progression (subproject 8644: Project Leader: Treloar Padovano), examination of psychopharmacological effects of oxytocin on stress-induced craving and the endogenous opioid system (P20GM130414; Project Leader: Haass-Koffler), and an experimental study of neural and immune effects of acute alcohol in people living with HIV (subproject 5261; Project Leader: Monnig). This mini-review focuses on the topic of liver health in the context of behavioral cofactors, using CADRE research projects as specific examples where applicable. Our aim is to illustrate the current challenges and opportunities in clinical research and care addressing liver health in the context of behavioral and medical comorbidity and to highlight next steps in this important line of work.

## 2 Clinical research examples on ALD and liver disease cofactors

### 2.1 ALD and alcohol use disorder (AUD)

Alcohol abstinence remains the best-known treatment for ALD. Helping individuals reduce drinking prior to developing advanced disease and shaping public policy to reduce drinking at a population level are the only preventative interventions available. Many ALD patients struggle to achieve alcohol abstinence due to concurrent AUD, which is itself a chronic condition characterized by alcohol craving and relapse (18–20). AUD rates are disproportionately rising among those at greater risk for ALD mortality. Cofactors modifying ALD progression include genetic, environmental, and behavioral risks, including biological sex, ethnicity, AUD severity, obesity-promoting behaviors, and smoking. Biological females have higher metabolic sensitivity to alcohol and develop ALD at lower alcohol-consumption rates and progress more quickly to end-stage liver disease than biological males (7). Available comparative epidemiologic data suggest that Hispanic or Latine ethnicity is linked to higher rates of hepatic steatosis and accelerated disease progression in both ALD and nonalcoholic steatosis (21, 22). Ethnic differences in disease susceptibility may be related to higher obesity prevalence in Hispanic and Latine communities (23). However, females and persons with minoritized racial and ethnic identities have been drastically underrepresented in clinical studies of ALD.

One of CADRE’s major research projects sought to identify overlapping biobehavioral pathways in ALD and AUD. While numerous studies demonstrate effectiveness of behavioral interventions for AUD, surprisingly few clinical trials have tested their efficacy in ALD patients, and the rigor of extant studies is limited by primarily male, nondiverse samples (24, 25). We partnered addiction scientists and hepatologists to test the efficacy of a brief motivational interviewing, alcohol-focused intervention among ALD patients with AUD and a comparative sample of individuals with AUD who had not progressed to advanced ALD, per blood diagnostic biomarkers.

A main behavioral outcome was alcohol craving, a validated predictor of treatment response in AUD clinical trials. A gold-standard laboratory alcohol cue reactivity paradigm (26) was paired with ecologically valid assessments of alcohol cues and craving in daily life. Visual graphs of the in-daily-life craving assessments were combined with personalized liver health feedback as part of the brief motivational intervention. Aligning with the CADRE theme, this research aimed to explore biomarkers of inflammation and immune activation as mechanisms of persistent drinking in AUD and ALD. Specifically, the study sought to explore associations of systemic biomarkers of inflammation with laboratory and real-world alcohol craving, toward the ultimate goal of a more holistic understanding of treatment nonresponse.

A primary aim was to demonstrate feasibility of enrolling ALD patients in the brief motivational alcohol intervention from routine clinical care. Common barriers to recruiting ALD patients in the clinical care setting soon became apparent (e.g., high clinic no-show rates, medical comorbidity, and disease severity), illustrating, in part, context for the lack of clinical trials testing behavioral AUD treatments in this population. Institutional structural barriers and lack of integration with local hospital systems also impeded study progress. We pivoted, and the soon-to-wrap-up study will meet overall recruitment targets through shifting the focus farther upstream in the ALD progression. Recruitment via social media successfully reached a diverse participant population of heavy drinkers with AUD at risk for ALD.

Offering the study in Spanish was essential to recruiting individuals who identified as Hispanic or Latine. Since its inception, our study aimed to achieve equitable representation of biological females and individuals who identify as Hispanic or Latine. All study materials, laboratory procedures, and the intervention manual were implemented in English or Spanish, thereby not excluding monolingual Spanish speakers from the research. Additional research costs incurred by these activities does not outweigh the need or benefit.

### 2.2 ALD and human immunodeficiency virus (HIV) infection

Another major project of the CADRE investigates the gut-liver-brain axis in people living with HIV infection (PLWH). Liver disease is a leading cause of death in PLWH, accounting for 13–18% of all mortality (27–29). Investigating effects of alcohol on the liver in the context of HIV is key because PLWH show higher levels of advanced liver disease compared to seronegative individuals, even at nonhazardous levels of alcohol consumption (30). Using experimental alcohol administration procedures, this study uses a 2×2 design (i.e., alcohol vs. placebo beverage; HIV seropositive vs. seronegative participants) to examine the effects of an acute, moderate dose alcohol on biomarkers of gut, liver, and brain health.

Outcomes include peripheral biomarkers of gut- and liver-mediated inflammation, as well as magnetic resonance imaging (MRI) measures of cerebral metabolism. For example, the study quantifies alcohol-induced change in soluble cluster of differentiation 163 (sCD163), an acute phase protein that reflects activation of liver macrophages, i.e., Kupffer cells, in response to inflammatory stimuli, such as alcohol or endogenous molecules (31, 32). In heavy drinkers, alcohol consumption shows a positive linear relation with sCD163 (33). In addition, sCD163 performs well as a marker of liver damage in individuals with HIV (34, 35).

This ongoing project is based in Providence, Rhode Island, and recruits PLWH from the largest outpatient provider of HIV care in the state. Despite integration of recruitment efforts into this clinical setting, however, enrollment of PLWH has presented a major challenge to this study. Barriers to enrollment of PLWH into liver-focused research include both structural and study-related issues. Structural issues include access to clinic patients during busy visits and high patient no-show rates. Lack of shared research administration between the academic institution and hospital system is a major barrier that many clinical researchers face in conducting biobehavioral studies. Study-specific barriers include narrow eligibility criteria needed to ensure safety for study procedures, i.e., alcohol administration, blood draw, and MRI. Individual-level issues include unwillingness of patients to be approached for any research purpose; transportation; and scheduling availability around other obligations such as work. Solutions to these problems have been offered to the extent possible, such as provision of paid transportation. In addition, the study has implemented alternative methods of recruiting PLWH, including social media and targeted ads, resulting in an improved rate of enrollment. Our experience suggests that reaching out directly to individuals in the community was more successful than clinic-mediated recruitment for this specific study on alcohol and liver disease in PLWH.

### 2.3 ALD and obesity

Metabolic dysfunction-associated steatotic liver disease (MASLD) is the most prevalent liver disease globally and affects ~25% of the US (2, 36). Obesity is the most significant risk factor for MASLD, affecting ~80% of persons with obesity (36). Obesity and alcohol use synergistically increase risk of liver damage, leading to cirrhosis, liver cancer, and death from liver disease (37–39). Current consensus is that that any alcohol use can accelerate progression of MASLD (40). Metabolic syndrome and excessive drinking independently increase risk of mortality in individuals with MASLD or ALD (41). Yet biological and behavioral factors mechanisms leading to steatosis (i.e., fatty liver) and fibrosis (i.e., scarring) in individuals with obesity and at-risk drinking is unclear (42, 43).

Despite clear overlap in behavioral precedents and adverse health outcomes, the fields of obesity and substance use research historically had minimal crosstalk or collaboration, and this disjunction is apparent in research on liver-health consequences. Thresholds for “moderate drinking” differ between the American Association for the Study of Liver Disease (40), the National Institute for Alcohol Abuse and Alcoholism (44), and other US and international research groups. Such differences are not trivial and make interdisciplinary research challenging. Clinically, the distinction between ALD and MASLD is made by self-reported alcohol use. As acknowledged by the American Association for the Study of Liver Diseases (40), the alcohol-use threshold used to differentiate ALD and MASLD in clinical practice is arbitrary and, in reality, few patients fit neatly into one category (42). Consequently, prior research treated MASLD and ALD as separate entities, despite shared pathophysiology and the co-prevalence of overweight/obesity and at-risk drinking (42). Notably, expert consensus published in December 2023 designates a new category of chronic steatotic liver disease termed metabolic and alcohol-associated liver disease, abbreviated MetALD (45). This new MetALD diagnostic category reflects the clinical reality that there is a significant proportion of individuals with both overweight/obesity and at-risk alcohol intake as contributors to their chronic liver disease. This newly recognized group is understudied and undertreated. An approach that integrates obesity, hepatology, and alcohol expertise is needed to address this clinical reality while optimizing benefits to patient care and public health.

## 3 Advancing research on behavioral cofactors and conditions in liver health

Studies to characterize risk for liver disease in individuals as a function of concomitant risk factors such as ALD, HIV, and obesity are critical for development of novel individualized lifestyle and liver-health interventions to reduce risk. Key goals are to identify: (1) the most effective ways to reach individuals with concomitant liver health risk factors and engage them in research and screening; (2) pragmatic, non-invasive approaches to liver screening and their contribution to early identification and behavior change; (3) novel biomarkers that could be used in future trials focusing on these conditions; and (4) factors that contribute to the interplay between alcohol use and lifestyle behaviors that promote risk for liver disease. Elements of special emphasis for public health benefit include: (1) integrating rather than dichotomizing alcohol use and risk cofactors, e.g., HIV or obesity, in liver disease; (2) focusing on representation in liver health research, particularly women and individuals of minoritized racial or ethnic identities; (3) screening early to identify signs of liver damage in specific high-risk populations.

As noted, efforts to recruit individuals to participate in clinical research on liver health often rely on medical record reviews and recruitment from local hepatology, gastroenterology, or other specialty clinics. Limitations of this strategy include the extremely high no-show rates coupled with low interest or ability to participate and comply with research protocols. These barriers are due, in part, to the nature of chronic and complex intersecting behavioral health conditions (e.g., AUD), comorbid health conditions (e.g., HIV infection), and liver impairment. Community-based engagement strategies would improve the ability to meet the target population where they are, toward the goal of engaging the broader population of adults with poor liver health who are not linked with treatment or not engaging with treatment. The goal is to enhance research access for adult populations that: (1) are historically difficult to engage in clinical care and in research studies; (2) experience social determinants of health (e.g., location, resources, education) as key barriers to research participation; (3) are at high risk of liver-related health problems from alcohol use, opioid use, obesity, etc.; and (4) are in high need but low receipt of intervention to reduce risks to liver health.

Ideally, integrated care teams with psychiatrists, psychologists, social workers, and hepatologists would see patients in the same clinical setting (46). Simply having one appointment in one place would address a range of logistical barriers to care, such as transportation, as well as stigma-related barriers that place the burden of finding AUD treatment on the ALD patient. Ideally, researchers would adopt a team-science approach to draw from strengths of siloed institutions for multisite studies and consider remote digital monitoring and telehealth approaches to reach populations who are too medically compromised to attend study visits.

Approaches to lessen provider, patient, and systemic/administrative barriers include: (1) expanded medical training curriculum using evidence-based models; (2) provider education in brief screening tools and screening, brief intervention, and referral to treatment (SBIRT) models; (3) training in motivational interviewing style, e.g., partnering and empathizing with the patient, and specific techniques to promote lifestyle changes, e.g., selectively reinforcing “change talk” in favor of goal behavior while softening “sustain talk” in favor of unhealthy behavior; (4) applying digital health monitoring and telemedicine approaches to deliver interventions to those who are too ill to attend appointments; (5) involving social work support to address financial and resource barriers.

Detecting disease modifiers is essential to appropriately allocate preventative interventions and apply personalized treatment. Ethnic disparities in chronic liver disease are complex, with likely contributions from genetic and environmental factors, as well as inequities in health-care access and socioeconomic wealth (22). Liver health disparities are likely to intersect with disparities in the treatment cascade for comorbid conditions such HIV infection. As one such example, Black individuals living with HIV have a lower likelihood of initiating antiretroviral therapy and attaining viral suppression (47–49). Comparative epidemiologic studies among specific ethnic groups are sorely needed, and the potential intersection of sex, ethnicity, and lifestyle cofactors in modifying disease risk needs to be explored. Such studies are key to understanding where along the ALD spectrum disparities begin to emerge and which risk determinants differ at each stage.

Chronic liver disease driven by lifestyle factors does not develop overnight. And yet, patients are expected to stop drinking overnight when told by their care provider to do so. Similar frustrations may be experienced by patients with obesity or diabetes who are advised to improve their eating habits or increase their physical activity. Although new biologic prevention and treatment options for ALD are sorely needed, effective behavioral and pharmacological options to treat concurrent AUD are currently available but underutilized. Evidenced-based interventions for AUD, such as screening and brief motivational interviewing or acamprosate treatment, are as effective as many other common medical interventions, such as statins for high blood pressure. However, AUD treatments are understudied and underutilized with ALD patients. Moreover, referral bias, exclusive focus on advanced disease stages in a clinical care setting, non-standardized disease definitions, and accuracy of screening and diagnosis tools are well-documented limitations in research and treatment. There is a high need for training ALD medical providers in AUD screening, diagnosis, and brief motivational approaches.

## 4 Conclusion

The CADRE’s experience conducting biobehavioral research on liver health in the context of chronic diseases, specifically AUD and HIV infection, is just one powerful exemplar of the need for integrated liver healthcare and research. Establishing multidisciplinary care clinics is a critical next step to better understanding and addressing the intertwined pathophysiology of ALD and chronic diseases through a collaborative team of hepatologists, pharmacists, addiction psychologists, and psychiatrists. Models for integrative ALD care clinics have been proposed, and real-life examples include the Delivery of Early Liver Transplant for Alcoholic Hepatitis (DELTA) Center for Alcohol Research at Johns Hopkins University and Michigan Alcohol Improvement Network (MAIN) at the University of Michigan (46, 50–52). The overlap of ALD with chronic, behaviorally driven conditions such as obesity, heart disease, and diabetes is an understudied issue of massive and increasing significance in the US population, pressing the need to disseminate effective models for integrated research and care.

## Author contributions

MM: Conceptualization, Writing – original draft. HT: Conceptualization, Writing – original draft, Writing – review & editing. PM: Conceptualization, Writing – review & editing.
